# Quantification of adaptive forces on SNP rs1010211 due to viral zoonotic pathogens

**DOI:** 10.1007/s10867-022-09606-y

**Published:** 2022-04-15

**Authors:** Daniah Alsufyani, James Lindesay

**Affiliations:** 1 College of Sciences and Health Professions, King Saud bin Abdulaziz University for Health Sciences, Jeddah, Saudi Arabia; 2 King Abdullah International Medical Research Center, Jeddah, Saudi Arabia; 3 Computational Physics Laboratory, Department of Physics, Howard University, 2355 Sixth Street NW, Washington, DC USA

**Keywords:** Adaptive force, Population health, SNPs, Genodynamics, Environmental parameters, Environmental homeostasis

## Abstract

Widespread genotyping of human populations in environmental homeostasis has created opportunities to quantify how environmental parameters affect the genomic distribution of variants in healthy populations. This represents an ongoing natural experiment upon the human species that can only be understood through developing models of adaptation. By examining the information dynamics of optimal SNP distributions within such populations, “adaptive forces” on genomic variants can be quantified through comparisons between different populations. To this end, we are performing double-blind scans of SNPs in order to ascertain any potential smooth functional relationships between the frequencies of the variants and changes in quantified environmental parameters. At present, we have sequentially examined more than twenty thousand SNPs (on chromosome 3) of nine homeostatic native populations for potential adaptive flagging of the variants as functions of 15 environmental parameters. Our first significant flag has related rs1010211 to viral pathogens in mammalian hosts. Such pathogens present a significant risk for the emergence of new infectious diseases in humans. This genomic variant is within the gene TNIK, which is a germinal center kinase (GCK). GCKs are participants in both adaptive and innate immune regulation. However, the function of TNIK is not fully understood. We quantify the adaptive force on the C allele due to the pathogens as 0.04 GEU’s/viral species.

## Introduction

The increasing availability of population-based data on genomic variants has inspired the development of several analytic tools to aid in our understanding of associations between those variants and biological and environmental factors. For example, genome wide association studies (GWAS) have been used to develop associations between specific genetic variations and particular diseases. Computational and statistical genomics combines statistics, epidemiology, mathematics, molecular genetics, and computer science to identify genomic variants associated with increased susceptibility to disease and variation of phenotypic traits. Precision health research in genomics utilizes large-scale informatic methods to examine both inherited disorders, as well as those that are due to new genetic mutations. Typically, the tools utilized in bio-informatics carry no dimensional units analogous to those used to quantify the dynamics of physical systems.

Novel applications of statistical physics to evolutionary biology [1] derive an analogous free energy function for the evolutionary dynamics of finite populations. An important simplifying assumption is that mutations are sufficiently rare that they are not affected by other segregating alleles. The formulation presented here is *not* about survivable mutations, which necessarily take a population temporarily out of homeostasis. In the absence of temporal genomic information over many generations, little can be inferred about the *rates* of evolutionary adaptation of the genome.

On the other hand, genodynamics quantifies the information *dynamics* of bi-allelic single nucleotide polymorphisms (SNPs) as functions of independent (usually environmental) variables, assuming that populations in homeostasis have optimized *overall population health*. Using concepts in evolutionary biology, a perturbed population in homeostasis evolves toward fitness peaks for the allelic variants [2]. For our formulation, any survivable genomic mutation that can be passed to offspring *inherently* takes the population slightly away from *adaptive* homeostasis until that modification distributes itself throughout the entire population. For this reason, only *common* variants are utilized to examine purely adaptive, non-evolutionary homeostasis in populations. One should not confuse the homeostasis of *stationary* systems with thermodynamic equilibrium. Living systems (and populations) are far from equilibrium, yet they maintain particular measurables associated with healthy homeostasis.

Our approach differs from standard bio-informatics in the use of *universal* dynamic genomic energy units (GEUs) that allow direct comparisons between different populations. Once such units have been established, any mathematically smooth relationship between dimensional allelic variants and quantified independent variables define adaptive “forces” between populations associated with migrations between those environmental differences. It should be emphasized that these adaptations optimize population health, absent a focus on disease.

## Methods

Human populations in environmental homeostasis are assumed to maintain a distribution of genomic variants that enhances the overall health and survival of that continuing stable population. Certain environmental agents (like malarial vectors) require a high degree of variation in certain alleles which can affect *individual* health in both beneficial and detrimental ways. Other agents (like UV-B radiation) elicit a change from one set of highly conserved variants to another for populations residing in the environmental extremes. In an effort to model the maintained frequencies of genomic variants within a population in Hardy–Weinberg equilibrium, an approach that describes the dynamics of populations in homeostasis within stable environments should be developed. Living systems in homeostasis are far from being in *thermal* equilibrium with indigenous environmental state variables (e.g., air temperature, air pressure, etc.). However, the modeling of many biophysically stationary processes in living systems is a goal of systems biology. Similarly, a description of generationally stable human *populations* should likewise, on some scale, be representable using macroscopically stationary formulations. In most instances, mathematical stability can be represented in terms of functional dependencies that remain unchanged under minor perturbations in the dynamic variables of the system (i.e., derivatives vanish near stationary values). Therefore, variables of state analogous to those of thermodynamics have been developed [3, 4] to describe the frequencies of genomic variants within homeostatic populations in an environment with persistent, yet common, agitations and stimulations upon populations absent significant survivable mutations.

Physical processes can be mathematically quantified once dimensional units have been developed. One can associate a dynamical genomic *unit* with the degree of variation, having a minimal value in populations with a universally conserved allele, and higher values based on the degree of variation within the population. For our purposes, a standard unit of 1 GEU (genomic energy unit) will be defined as that degree of environmental agitation that invokes maximum variation (50–50%) of the alleles in a bi-allelic SNP that is not in linkage disequilibrium with other SNPs.

The development of a standard measure of variation provides a *human*-universal dimensional unit that allows comparisons of the relative degree of “forcing” or “pressure” that a given quantifiable environmental agent has on genomic variation (in a manner analogous to tools utilized in physical sciences). For instance, in physics, a force is defined in terms of the gradient down the slope of an energy curve. In analogy, once dimensional genomic potentials ${\mu }_{a}$ can be assigned to alleles, adaptive forces can be defined as the gradients of those potentials,

1 $${f}_{a}=-\frac{\partial {\mu }_{a}}{\partial \lambda }$$

if the potentials vary with environmental parameters $\lambda$ using smoothly differentiable functional forms.

Populations that reside in environments with more robust agents of influence exhibit a higher degree of variation than those residing in environments with fewer stimulants and pathogens. The overall intensity of genomic stimulation due to environmental agents can be quantified in terms of the degree of variation (i.e., disorder) of the genome. The degree of disorder of variants in a single biallelic SNP not in linkage disequilibrium is expressed in terms of its specific entropy

2 $${s}^{(S)}=-\sum_{a=1}^{2}{P}_{a}^{\left(S\right)}{\mathit{log}}_{2}{P}_{a}^{\left(S\right)}$$

where ${s}^{(S)}$ is the entropy per individual of a single bi-allelic SNP (*S*), and ${P}_{a}^{(S)}$ is the probability of occurrence of allele $a$ in the homeostatic population. A similar equation can be developed for the entropy of a *haploblock* of linked SNPs [3]. As defined, entropies are additive state variables quantified on contiguous regions of the population’s genome.

The overall degree of genomic disorder in a population sums over the non-linked as well as haploblock entropies, ${s}_{Genome}=\sum_{S}{s}^{(S)}+\sum_{H}{s}^{(H)}$. A relative degree of *order* (i.e. maintained information) can be defined in terms of the normalized information content (NIC) given by

3 $${NIC}_{genome}=\frac{{S}_{max}-{S}_{genome}}{{S}_{max}}$$

This normalized measure of maintained *order* varies between 0 and 1, allowing comparisons of distributions of variants for different regions of the genome, and between differing populations. A *lower* value of NIC is indicative of a more disordered distribution. The NIC is analogous to the *evolutionary information density D(N)* = *1-S/N* found in the literature [5] since for biallelic SNPs, *s*_*max*_ = *N*_*SNPs*_.

For our purposes, only native populations in environmental homeostasis will be examined. For thermodynamic systems in thermal *equilibrium* (analogous to *homeostasis*), the Helmholtz free energy *F* is minimized *(dF* = *0*), where

4 $$dF=- SdT-PdV+\sum {\mu }_{j}d{N}_{j}$$

The temperature T quantifies an intrinsic (intensive) degree of agitation imposed upon the system due to the thermal bath. The analogous form for the genomic free energy associated with a homeostatic population in an environmental bath is given by

5 $$d{F}_{Genome}=- {S}_{Genome}d{T}_{E}+{\sum }_{S}{\mu }^{(S)}d{N}^{(S)}+{\sum }_{H}{\mu }^{(H)}d{N}^{(H)}$$

once allelic potentials $\mu$ can be quantified. In this expression, the genomic entropy *S*_*Genome*_ is a population’s specific entropy times its size (*S*_*Genome*_ = *N*_*population*_* s*_*Genome*_*.*).

For bi-allelic SNPs not in linkage disequilibrium, the allelic potentials are given by

6 $${\mu }_{a}^{\left(S\right)}=\left(\stackrel{\sim }{\mu } -{T}_{E}\right)-{T}_{E}{\mathit{log}}_{2}{P}_{a}^{(S)}$$

where $\stackrel{\sim }{\mu }$ = 1 GEU. The form is chosen so that these potentials have units of GEUs that are additive when the probabilities are independent (i.e., multiplicative), and a potential takes the standard value of 1 GEU when there is maximum variation *P*_*a*_ = *1/2.*

Given Eq. (5), the value of *T*_*E*_ for a specific population can be determined as a measure of environmental agitation analogous to the temperature of a thermal bath in thermodynamics. A system in thermal *equilibrium* with uniform temperature T has its Helmholtz free energy minimized. In analogy, the *genomic* free energy of a population in *homeostasis* will be stationary. By optimizing the overall genomic free energy relative to total population size *N*_*population*_, ${\left(\frac{{\delta F}_{Genome}}{{\delta N}_{population}}\right)}_{{T}_{E}}=0,$ one establishes an expression for the genodynamic parameter ${T}_{E}$ conjugate to genomic entropy (which increases with degree of disorder),

7 $${T}_{E}=\frac{\stackrel{\sim }{\mu }}{{NIC}_{genome}}$$

as well as the optimal population size relative to the environmental resources.

This formulation, therefore, requires the calculation of the *whole*-genome information content for bi-allelic variants within each population to be explored, which involves a substantial computational effort. Previous explorations focused on the HapMap populations for which *all* variant information has been calculated [3]. Rather than duplicating this for the substantially larger number of variants and populations in the 1000 Genome Project, we examined the HapMap data to determine a specific *single* chromosome whose normalized information content (NIC) most closely matched that of the whole genomes of the HapMap populations. Chromosome 3 was determined to be the best candidate (well within our < 1% acceptability criterion) [4]. Thus, in this study, the information content of the whole chromosome 3 of the examined populations using 1000 Genome data (which still remains a substantial effort) has been determined as an accurate measure of the overall environmental potential *T*_*E*_.

We have chosen populations that have likely remained in homeostasis with their ancestral environments to flag functional relationships of variant occurrences with quantified environmental parameters. For this study, we are utilizing the wealth of information available in the whole chromosome 3 data needed to calculate *T*_*E*_, and have chosen to perform a *double-blind* examination that flags potential relationships of SNP frequencies with a catalog of tabulated environmental parameters associated with each population. The populations chosen for this study were Peruvian in Lima, Peru [PEL], Colombian in Medellín, Colombia [CLM], Han Chinese in Beijing, China [CHB], Finnish in Finland [FIN], Kinh in Ho Chi Minh City, Vietnam [KHV], Toscani in Italy [TSI], Yoruba in Ibadan, Nigeria [YRI], Mende in Sierra Leone [MSL], and Iberian populations in Spain [IBS]. Fifteen ancestral environmental parameters have been quantified for each population, including altitude, temperature, rain, bacteria, virus, protozoa, helminth, UVB, wind speed, humidity, pressure, and pathogens from chiroptera, primates, rodentia, soricomorpha. The data was population-averaged using cities distributed in ancestral geographic regions. For non-tabular data (maps), the authors independently ascertained the quantified scales associated with the cities, and the averaged results were utilized. This effort is expected to take about a year of computational time to complete.

For further clarity to the reader, the various genomic potentials and parameters for a particular example will be demonstrated. First, consider the normalized information contents (*NIC*s) from Eq. (3) calculated using the entropy of the whole of chromosome 3 (which mirrors that of the whole genome) for the populations CHB (*NIC* = 0.90) and MSL (*NIC* = 0.81). The NIC varies from a minimum value of zero for a completely disordered genome, to a value of one for a completely homogeneous population of clones. This indicates that the genomes of the genotyped MSL population in Sierra Leone had a higher degree of variation than those in the CHB population in China. The parameters *T*_*E*_ (which quantify the overall degrees of *intensive* environmental agitation on the populations) conjugate to the genomic entropies *S*_*Genome*_ of the two populations are *T*_*E*_^*CHB*^ = 1 GEU/0.90 = 1.11 GEUs, and similarly *T*_*E*_^*MSL*^ = 1.23 GEUs.

Next, consider a particular population for which the alleles in a biallelic SNP (for instance A and G) occur with equal likelihood *p*_*A*_ = *p*_*G*_ = 1/2. Equation (6) then defines a universal unit of maximum environmental agitation for a SNP not in linkage dis-equilibrium, yielding genomic potentials ${\mu }_{A}$ = ${\mu }_{G}$ = 1 GEU. In contrast, for a different population homogeneous in G, *p*_*G*_ = 1 and ${\mu }_{G}$= (1 GEU – *T*_*E*_) = ${\mu }_{fixing}$ (which is the *fixing potential* of that population), while *p*_*A*_ = 0, generating a large genomic potential ${\mu }_{A}$ associated with the finite size of the sampled population.

Finally, consider a set of allelic potentials ${\mu }_{G}$ for the SNP in the previous paragraph for various populations as demonstrated in Table 1.

**Table 1:** Example values of the allelic potentials of the G allele (in GEUs) for a hypothetical SNP for various populations

Population	λ in meters	${\mu }_{G}$
PEL	1101	−0.1 GEUs
CLM	1005	0.9 GEUs
CHB	897	2.0 GEUs
FIN	801	2.9 GEUs
KHV	696	4.1 GEUs
YRI	598	5.1 GEUs
IBS	502	5.9 GEUs
TSI	399	7.0 GEUs
MSL	301	8.1 GEUs

**Fig. 1:**
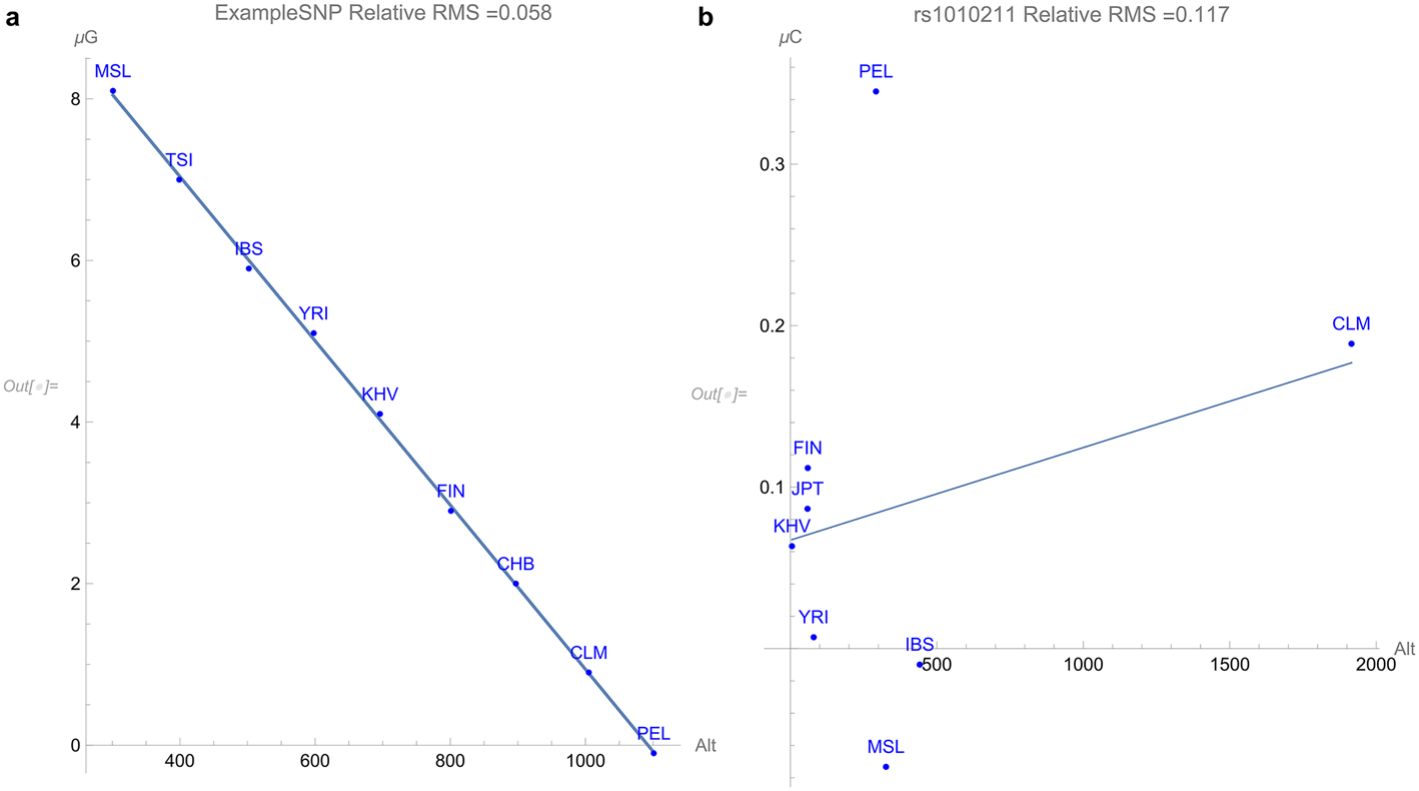
(**a**) An example plot of the allelic potentials of the G allele (in GEUs) for a hypothetical SNP versus altitude above sea level in meters. The slope of this curve indicates an adaptive force of + 0.01 GEUs per meter. (**b**) An actual plot of the allelic potentials of the C allele (in GEUs) for rs1010211 versus altitude above sea level in meters. This plot does not satisfy our threshold criterion for flagging an adaptive force

A plot for the allelic potential of the G allele as a function of the environmental parameter altitude is shown in Fig. 1a. The plot demonstrates an adaptive force toward increasing altitude of 0.01GEUs per meter, or 10 GEUs per kilometer of altitude. It should be emphasized that the allelic variation for *every* population optimizes the overall population health for that particular altitude. As an example of an environmental parameter that does not flag for an adaptive force, the response of the SNP to be later discussed is plotted against altitude in Fig. 1b.

## Results

**Fig. 2:**
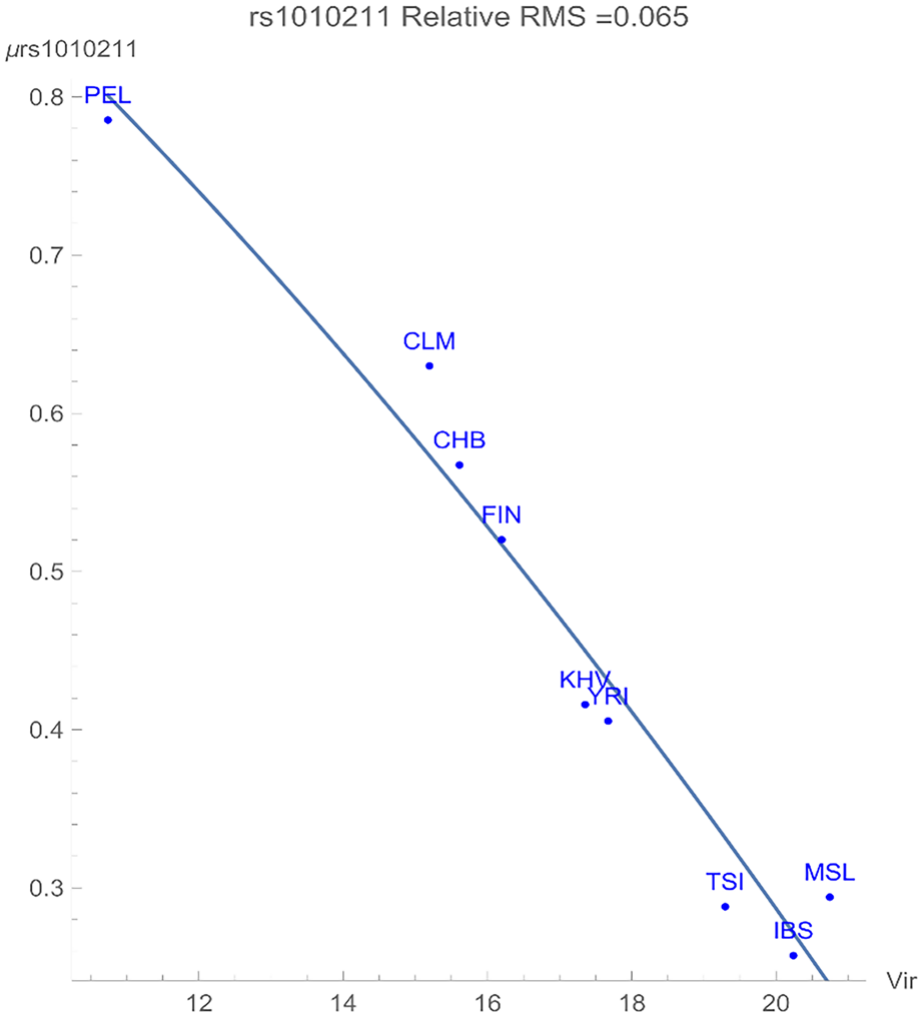
The correlation between the rs1010211 genomic potential values (in GEUs) and the richness (numbers of species) of zoonotic viral pathogens. The adaptive force is about 0.06 GEU’s/viral species

The SNP rs1010211 has been the only variant among the first twenty thousand on chromosome 3 to flag for a simple mathematical dependency on one of the fifteen environmental parameters. For this study, only simple adaptive dependencies with allelic potentials that are monotonic in the environmental parameters are flagged. Since the determination of an adaptive force requires a calculation of the derivative of a smooth functional form, only parameterized quadratic forms of the genomic *potentials* themselves, or parameterized forms resulting from simple dependencies of the *frequencies* of occurrence of the alleles themselves, were optimized for each dataset. A dataset was flagged only if the root-mean-squared deviation of the data points from the fitted curves was less than 10% of the maximum variation in the potentials. This polymorphism *only* flagged a relationship between zoonoses caused by viral pathogens in mammal groups (including carnivores, bats, primates, rodents, shrew, moles, and hoofed) [6] and allelic frequencies within the nine populations, as demonstrated in Fig. 2.

Figure 2 exhibits a direct dependency of the rs1010211 SNP potential expressed in genomic energy units (GEUs) upon the environmental virus richness pattern. Virus richness pattern is defined in the data source [6] as follows:

“Richness: the number of unique species within a particular geographic area; richness is a count-based metric for quantifying diversity, which contrasts with other metrics, such as functional trait diversity (the different types of traits represented within a geographic area) or genetic diversity.”

The SNP flagged a relationship with a positive adaptive force of about 0.06 GEU’s/viral species within a relative root-mean-squared degree of uncertainty of about 0.065. As shown in Fig. 2, environments with maximal viral richness such as Spain, Sierra Leone, and Toscani are associated with higher degrees of SNP conservation. In contrast, maximum variation of the SNP was found among the Peruvian population, which resides in an environment with the least viral richness.

**Fig. 3:**
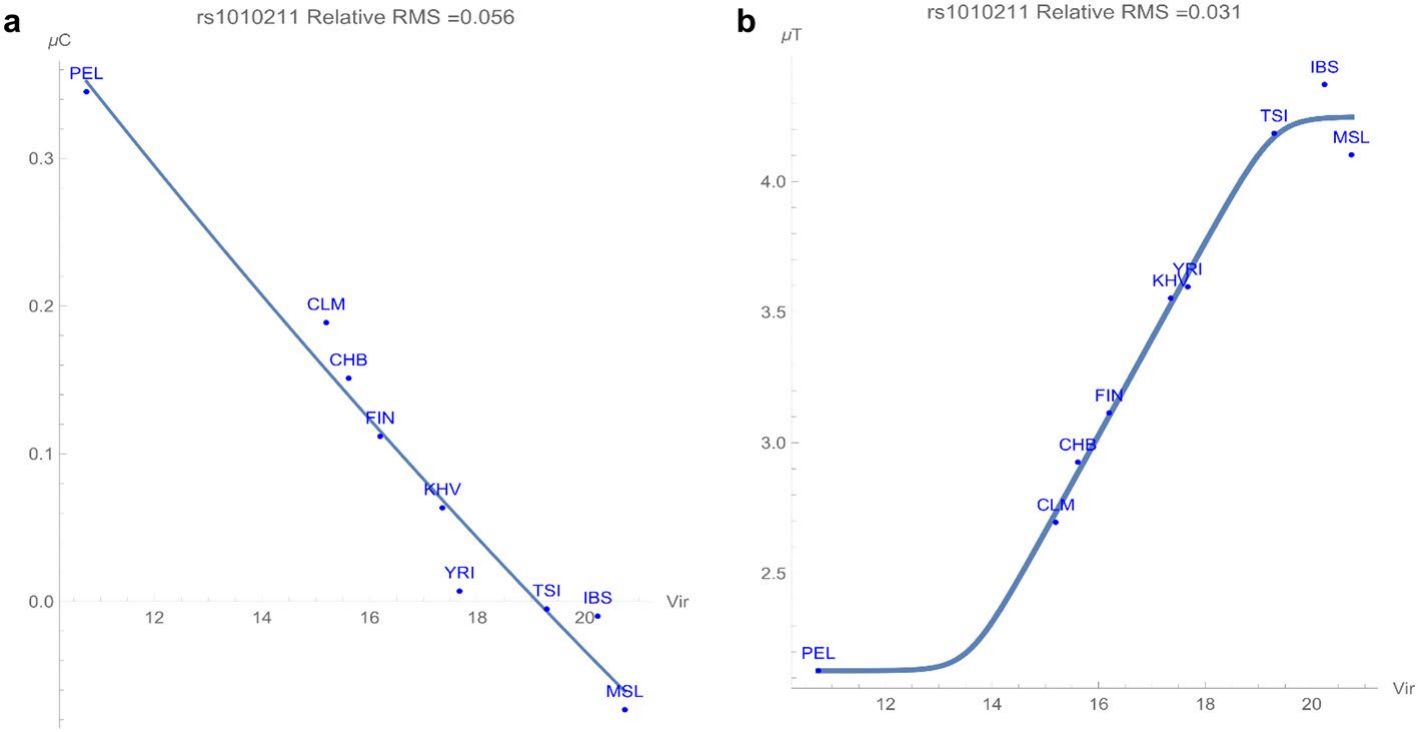
(**a**) The correlation between C allelic potential (in (GEUs) of rs1010211 and richness (numbers of species) of viral pathogens. The adaptive force is about 0.04 GEU’s/viral specie. (**b**) The correlation between T allelic potential (in (GEUs) of rs1010211 and richness of viral pathogens. The adaptive force is about −0.2 GEU’s/viral species

Plots for biallelic potentials must be consistent with the frequencies of the common variants adding to one within the whole population. The minor allele always has the higher genomic potential of the two variants. For rs1010211, the common alleles are T and C. The next graph, Fig. 3a, exhibits a relationship between the allelic potentials of the C allele and the viral pathogens, with an adaptive force of about 0.04 GEU’s/viral species. Figure 3a demonstrates that populations residing in areas with higher viral richness pattern have lower allelic potential (e.g., MSL is nearly homogeneous in C). In other words, the C allele is more conserved in populations that live in areas with high viral zoonoses (a positive adaptive force).

On the other hand, the allelic potential of the T allele in Fig. 3b has a direct relationship with viral zoonoses, with an adaptive force of more than −0.2 GEU’s/viral species. Populations living in regions with lower viral richness have lower T allelic potentials (i.e., higher frequencies of the T allele in this variant, a negative adaptive force) while those residing in regions with higher viral richness have higher allelic potentials. This opposing dependency of the two alleles on the virus distribution suggests a biologically simple relationship between the occurrence of the alleles in this SNP and the viral zoonoses.

## Discussion

During our double-blind search for adaptive influences on SNPs in chromosome 3, we are choosing a hard cutoff in the relative root-mean-squared deviation from fitted functional forms of 0.1 while examining fifteen reliable environmental data for all nine populations still residing in long-term ancestral environments.

All three allelic potentials of the rs1010211 polymorphism (i.e., each allele as well as the population-averaged overall SNP potential) *only* flagged for simple adaptive dependencies upon viral zoonotic diseases in mammals. Furthermore, each potential flagged *monotonically*, resulting in positive adaptive forces (increased conservation) for the SNP and C allele and a negative adaptive force for the T allele. It should be emphasized that *any* value of the allelic potential optimizes the overall health of a given population in homeostasis. The observation that all potentials flagged for this parameter is unusual and indicative of a simple biological association between this variant and mammalian viruses. It is particularly noteworthy that this SNP is not in linkage disequilibrium in *any* of the populations. In our experience, this is quite rare. Thus, the adaptive response to environmental stressors exhibited through this variant is likely relatively simple and universal among disparate human populations.

**Fig. 4:**
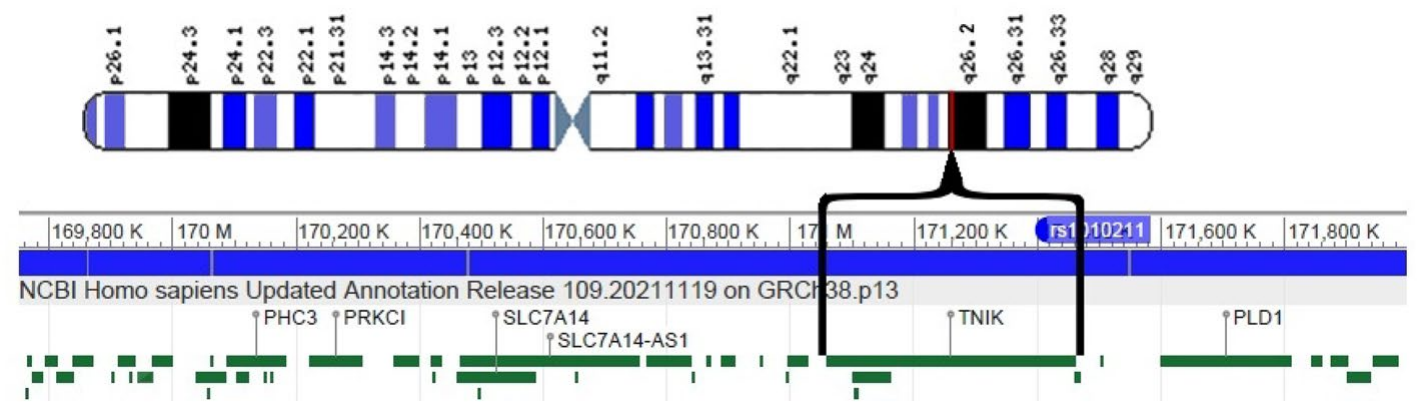
The map of the TNIK locus. Chromosome 3 has 199 million base pairs. The gene containing the flagged SNP, TNIK, extends from 171,058,414 to 171,460,408. The SNP rs1010211 is at locus 171,413,851

The flagged SNP rs1010211 is an intron variant in the gene TNIK, which is directly associated with adaptive immune response. This polymorphism is an ancestral variant common among the zoonotic mammals and humans [7]. Furthermore, this gene is *evolutionarily* conserved among various taxonomic classes (mammalia, aves, ray-finned fishes, amphibians). At least 197 organisms have orthologs (i.e., speciation with retention of function) with the human gene TNIK. The result that the flagged variant is shared between several taxonomic classes supports our statistical adaptation approach versus a nearly natural molecular evolution. As previously mentioned, this SNP is *not* in linkage disequilibrium with other SNPs in any of our populations, indicative of a relatively simple biological function, at least within the human populations. This function is likely shared among the various organisms. It has been identified with a protein that mediates the immune response in healthy B-cell. In a healthy cell, TNIK has been found to regulate not only immune response, but also cell division and cell death [8]. It furthermore regulates the immune system by activating B cells. It should be noted that B-cells function in the humoral immunity component of the *adaptive* immune system [9], where they produce specialized antibody molecules that then serve as a part of B-cell receptors [10]. It has recently been found that TNIK is also a regulator of effector and memory T cell differentiation, inducing a population of undifferentiated memory T cells [11]. Thus, it is not surprising that this variant would directly respond to viral zoonotic *adaptive* forces. A gene map of the TNIK locus is illustrated in Fig. 4.

TNIK is a member of the germinal center kinase (GCK) family involved in cytoskeleton organization and neuronal dendrite extension [12]. Germinal centers are transient structures within B lymphocytes found in secondary lymphoid organs (where the B cells differentiate and adapt their antibody genes during normal immune response to an infection) [13]. They play a crucial role in the humoral immunity component of the adaptive immune response which generates matured B cells that produce effective antibodies against infectious agents, as well as the production of durable memory B cells. Germinal center kinases participate in a variety of signaling pathways needed to regulate cellular functions including apoptosis, cell proliferation, polarity and migration [14]. They are involved in both adaptive and innate immune regulation. However, the differential activation and regulatory mechanisms of GCKs remain to be fully determined.

## Data Availability

HapMap and 1000 Genome data is open access. All environmental data used is freely available on line.

## Code availability

Not applicable, formulas can be coded using any programming platform.
